# Research Progress of Applying Infrared Spectroscopy Technology for Detection of Toxic and Harmful Substances in Food

**DOI:** 10.3390/foods11070930

**Published:** 2022-03-23

**Authors:** Wenliang Qi, Yanlong Tian, Daoli Lu, Bin Chen

**Affiliations:** 1 School of Food and Biological Engineering, Jiangsu University, Zhenjiang 212013, China; wenliangqi@stmail.ujs.edu.cn (W.Q.); tianyanlong11@163.com (Y.T.); dllu@ujs.edu.cn (D.L.); 2 Beijing Instrument Industry Group Co., Ltd., Beijing 100022, China; 3 Beijing Beifen-Ruili Analytical Instrument (Group) Co., Ltd., Beijing Engineering Research Center of Material Composition Analytical Instrument, Beijing Enterprise Technology Center, Beijing 100095, China

**Keywords:** infrared spectroscopy, toxic substance, chemometrics, research progress

## Abstract

In recent years, food safety incidents have been frequently reported. Food or raw materials themselves contain substances that may endanger human health and are called toxic and harmful substances in food, which can be divided into endogenous, exogenous toxic, and harmful substances and biological toxins. Therefore, realizing the rapid, efficient, and nondestructive testing of toxic and harmful substances in food is of great significance to ensure food safety and improve the ability of food safety supervision. Among the nondestructive detection methods, infrared spectroscopy technology has become a powerful solution for detecting toxic and harmful substances in food with its high efficiency, speed, easy operation, and low costs, while requiring less sample size and is nondestructive, and has been widely used in many fields. In this review, the concept and principle of IR spectroscopy in food are briefly introduced, including NIR and FTIR. Then, the main progress and contribution of IR spectroscopy are summarized, including the model’s establishment, technical application, and spectral optimization in grain, fruits, vegetables, and beverages. Moreover, the limitations and development prospects of detection are discussed. It is anticipated that infrared spectroscopy technology, in combination with other advanced technologies, will be widely used in the whole food safety field.

## 1. Introduction

With the rapid development of the food industry, China’s food safety level is constantly improving. However, it still faces many problems, such as illegal food additives and the abuse of antibiotics. The quality and safety of food involve everyone’s health and safety. Such problems not only exist in individual countries but also present as a global problem. For instance, “melamine” and the “Sudan red duck egg” event, among others, have brought fatal consequences [1,2]. According to the different sources of harmful substances, they can be divided into three kinds: endogenous, exogenous toxic and harmful substances, and microbial toxins. Endogenous toxic and harmful substances can be divided into endogenous toxins and toxic substances produced in processing. Endogenous toxins include toxic proteins, alkaloids, phenols in plant-derived agricultural products [3,4,5], tetrodotoxin, shellfish toxin, and so on, in animal-derived agricultural products [6,7]. Exogenous toxic and harmful substances mainly come from the external environment, and pollution to food, such as pesticides. They may occur in every link of the food supply chain [8], from the contamination of veterinary drugs [9], heavy metals and harmful elements [10], and from using food additives, antibiotics, dioxins, and their analogs. Microbial toxins consist mainly of bacterial and fungal toxins, such as cyanobacteria toxins, aflatoxin, etc. [11,12].

For different kinds of toxic and harmful substances, the commonly used detection methods are gas chromatography (GC), high-performance liquid chromatography (HPLC), gas chromatography–mass spectrometry combined technology (GC-MS), liquid chromatography–mass spectrometry (LC-MS), enzyme-linked immunosorbent assay (ELISA), etc. [13,14,15]. Most of these methods have high requirements for high-cost instruments and complex sample preprocessing. Infrared spectroscopy technology is widely used in the detection of toxic and harmful components in food with its fast, nondestructive, efficient, and convenient operation. This technology is also a common detection method in modern structural chemistry and analytical chemistry. The characteristics of the molecular structure can be distinguished according to the position and intensity of the infrared absorption peak. The absorption peak intensity of the spectra is positively correlated with its chemical group content. Therefore, it becomes the principal method for detecting toxic and harmful substances in food. In this review, the application of infrared spectrum technology in the detection of toxic and harmful substances in food is introduced, and its advantages and disadvantages in the detection process are discussed. It is expected that this paper will provide theoretical guidance for the analysis and detection of toxic and harmful substances in the food industry and agriculture.

## 2. Principles of Detecting Toxic and Harmful Substances

### 2.1. Principle of Infrared Spectroscopy Technology

Infrared spectroscopy is an analytical method for using intermolecular vibrations to identify molecular structures. When an infrared light source with a continuous wavelength radiates the measured object, the infrared light at a specific frequency is absorbed by a specific molecule corresponding to the characteristic bond in the sample molecule that selectively absorbs light from the infrared region of the electromagnetic spectrum, causing molecular vibration. The absorption specificity corresponds to the characteristic chemical bonds in the sample molecule. Generally, the infrared spectral interval is divided into three regions, namely, the near-infrared region (12,800~4000 cm^−1^), mid-infrared region (4000–400 cm^−1^), and far-infrared region (400–10 cm^−1^) [16].

### 2.2. Fourier Transform Infrared Spectrometer Principle

**Figure 1:**
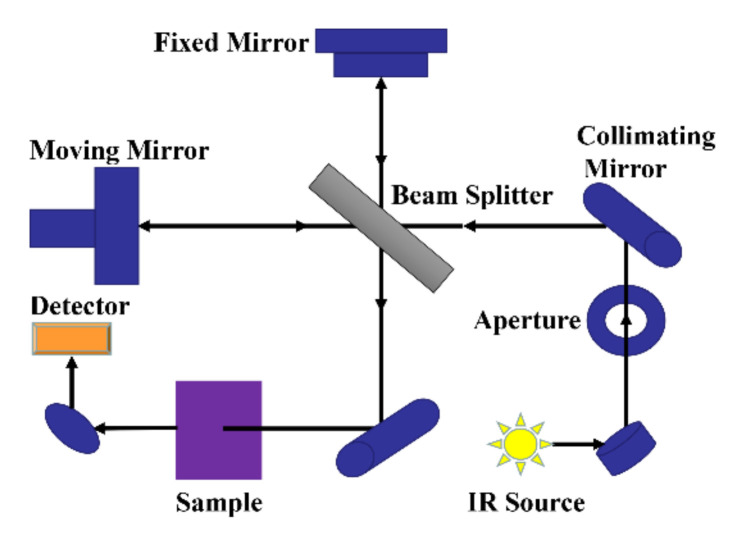
Schematic diagram of the optical system of the mid-infrared spectrometer.

Fourier transform infrared spectrometer is the mainstream instrument used for infrared spectroscopy analysis. Figure 1 is a schematic diagram of the optical system of the Fourier transform infrared spectrometer, mainly composed of the fixed mirror, moving mirror, beam splitter, appendix, light source, etc. The light from the light source reaches the beam separator through the collimated lens, which is divided into two beams: one beam through the transmission to the moving lens, and the other beam by reflection on the fixed mirror. Two beams of light are reflected by the fixed mirror and the moving mirror and return to the beam splitter. The moving mirror is moving at a constant speed, so the two beams of light through the beam splitter form the light range difference and produce interference. Interferometric light passes through the sample pool after the beam splitter meets, through which the interference light containing the sample information reaches the detector, and then processes the signal by the Fourier transform to finally obtain an infrared absorption spectrogram of transmittance or absorbance with a wavenumber or wavelength.

There are four main Fourier transform infrared detection modes: transmission, attenuated total reflection (ATR), mirror reflection, and diffuse reflection mode. Users can choose appropriate detection methods according to different detection needs and physical states of the sample. Solid samples can be detected by transmission spectroscopy, attenuation full reflection spectroscopy, etc. Liquid samples can be detected by transmission spectroscopy of an infrared liquid pool or reflection spectrum of attenuated full reflection accessories.

### 2.3. Principle of Near-Infrared Spectroscopy

Near-infrared spectroscopy (NIR) is an instrument detecting molecular vibration spectra ranging from 12,800 to 4000 cm^−1^. The near-infrared spectra mainly absorb C-C, O-H, C-H, N-H, S-H, etc. [17]. The double and co-frequency of group vibrations contain component information of most organic compounds. NIR spectroscopy is efficient, fast, nondestructive, and requires no sample pretreatment, being advantageous in the quality control of food, biological, medicinal, and chemical products [18,19]. Modern infrared spectroscopy is a fusion of spectral measurement, computer science, chemometrics, and basic detection [20,21].

In the qualitative analysis, the NIR spectrum is related to the chemical composition and content of the material itself, and the same chemical composition. The material structure and content of the sample determine the attribute characteristics of the sample. The NIR spectrum is used as a variable to establish the correspondence between the sample genus and the NIR spectrum, which is then applied to the NIR spectrum to calculate and obtain the genus or characteristics of the sample [22]. Changes in the sample composition in quantitative NIR spectral analysis have caused changes in sample properties and also changes in molecular spectra. There is a correlation between the sample composition concentration or properties and the corresponding molecular spectral changes. By establishing the correction model using a multivariate correction method and applying the model, unknown sample spectra can be obtained to achieve a quantitative prediction of single or multiple composition concentrations or properties [23].

## 3. Spectral Data Processing Technology

In the process of spectral acquisition using infrared spectroscopy, in order to improve the instrument’s signal-to-noise ratio, it is necessary to eliminate the interference of mixed stray light, noise, and other factors, separate overlapping peaks, and then extract useful information. The spectral data is usually combined with chemometrics. Spectrum combined with the mode recognition method can quickly and accurately identify the authenticity of the objects to be measured and improve prediction accuracy. Using multiple correction techniques, such as multiple linear regression (MLR), principal component regression (PCR), and partial least squares (PLS), can improve the accuracy and reproducibility of analysis tests and reduce background interference.

Valas et al. [24] used the Kubelka–Munk transform and first-order derivatives for the prediction of pistachios containing aflatoxin, and the discriminant analysis correctly separated 100% of the calibration and validation sets and 80% of the test set. Cebrián et al. [25] developed a support vector machine-discriminant analysis (SVM-DA) model approach for the prediction of ochratoxin A (OTA) in dry-cured ham with a prediction sensitivity of 85%. Freitas et al. [26] used a multilayer perception network (MLP) and partial least squares (PLS) to identify milk samples contaminated with tylosin below, equal to, or above the maximum residue limit (MRL) with an accuracy of >99%. Multi-curve resolution-alternating least-squares (MRL) was used by Mazivila et al. [27] to identify milk samples contaminated with tylosin with an accuracy of >99%. Mazivila et al. [27] applied multivariate curve resolution-alternating least squares (MCR-ALS) as a complementary chemometric model to the DD-SIMCA. Haruna et al. [28] used competitive adaptive reweighted sampling-partial least squares (CARS-PLS) for the quantitative determination of acid and peroxide values in peanut oil. Zaukuu et al. [29] used linear discriminant analysis (LDA) and partial least squares regression analysis (PLSR) to predict trace components, such as urea and melamine in whey protein powder. Ghidini et al. [30] developed a calibration model based on orthogonal partial least squares regression to predict the histamine content of tuna.

Chemometrics is a discipline that combines theories and methods from mathematics, statistics, computer science, and multiple disciplines to maximize useful chemical information that can be obtained from measurement data by optimizing the process of chemical measurements. Given that IR spectroscopy mainly reflects the chemical composition of the substance to be measured as a whole, standardized experiments must be strictly controlled in order to obtain high-quality and reproducible spectral data during specimen selection, sample preparation, and spectral acquisition. For liquid samples, transmission, and horizontal attenuation total reflection methods are mostly used for measurement. For solid and powder samples, diffuse reflection methods are mostly used for measurement. In order to obtain high-quality IR spectra and build more accurate models, chemometric methods are frequently used.

Spectral preprocessing is the processing or transformation of spectral data to reduce or even eliminate the interference of non-target factors in the spectrum and to improve spectral resolution and sensitivity. Commonly used chemometric methods are mainly smoothing, derivatives, multiple scattering correction, etc. After dividing the processed spectral data into sample sets, principal component analysis is used to extract the main characteristic components of the data. Qualitative model building is divided into supervised and unsupervised modeling. The supervised modeling is used to divide the data set first, start building the model after spectral processing, and evaluate the model with PLS-DA. The unsupervised modeling differs in that, after spectral processing, classification, and modeling are performed with PCA. For the prediction of quantitative models, the partial least squares method is mostly used. For the built prediction model, model testing is also needed to verify its prediction accuracy and sometimes model transfer is required.

## 4. Application of Infrared Spectroscopy Technology in Detection of Toxic and Harmful Substances in Food

### 4.1. Detection of Exogenous Toxic and Harmful Substances

During the production and processing of food or raw materials, contamination may be caused by various external causes and illegal human factors. The detection of such substances by infrared spectroscopy is of great importance.

Arzu Yazici et al. [31] established a method for rapid nondestructive detection of pesticide residues in strawberries based on near-infrared spectroscopy. By performing the second-order derivative, principal component analysis (PCA) on the spectral data, the prediction models for aminopyralid and azoxystrobin were developed using PLSR: the prediction correlation coefficient (*R_P_*) for aminopyralid was 0.93, the root mean square error of prediction (*RMSEP*) was 3.22 mg kg^−1^, and the relative prediction error (RPD) was 2.28. The prediction correlation coefficient (*R_P_*) for azoxystrobin was 0.91. Lu et al. [32] used visible-NIR spectroscopy combined with chemometric methods to quantify chlorpyrifos and carbendazim residues in cabbage. The quantitative models were developed using the partial least squares regression (PLSR) and least squares support vector machine (LS-SVM) methods. The feature variables were selected using the continuous projection algorithm (SPA), random frog algorithm, and PLSR methods. The LS-SVM model performed better than the PLSR model. The prediction correlation coefficient (*R_P_*) of chlorpyrifos samples was 1 and the root mean square error of prediction (*RMSEP*) was 0.03 mg kg^−1^. The average spiked recoveries ranged from 98.95–102.26% and the relative prediction errors ranged from 0.88–9.97% when the chlorpyrifos concentration was greater than 5 mg kg^−1^. The prediction correlation coefficient (*R_P_*) of chlorpyrifos samples was close to 1, the prediction mean-spiked recoveries ranged from 99.10% to 100.66%, and the relative prediction errors ranged from 0.39% to 5.01% when the concentration of carbendazim was greater than 1 mg kg^−1^, and the two samples had good consistency and reproducibility.

Liu et al. [33] investigated a method based on the radial basis function (RBF) neural network combined with NIR spectral data to predict the talc content in wheat flour. In this method, sample data were processed by multiple scattering correction, and the correlation coefficient method was used to reduce the spectral redundancy to determine the maximum relevant information wavelength. The prediction correlation coefficient (*R_P_*) of the optimal MSC-CCM-RBF model was 0.9999, the root mean square error of prediction (*RMSEP*) was 0.0765, and the relative prediction error (RPD) was 65.0909. Che et al. [34] used Vis-NIR reflectance spectroscopy based on physical property analysis to predict the concentration of azodicarbonyl in flour. An amount of 101 samples with a concentration gradient of 3 mg kg^−1^ were prepared using a stepwise dilution method in the concentration range of 0–300 mg kg^−1^. The abnormal samples were identified and rejected by combining the Marxian distance method and leave-one-out cross-validation, and the radial basis function model could better predict the concentration of azodicarbonyl in flour by selecting the characteristic band through correlation and using the preprocessing method of the first-order derivatives and SNV. The method yielded prediction correlation coefficients and the root mean square error of prediction (*RMSEP*) of 0.99996 and 0.5467, which were within 0.01 for each sample, and LOD and LOQ of 3.2 and 9.7 mg kg^−1^, respectively. Li et al. [35] used Fourier transform transmission infrared (FTIR) spectrometry to determine Pb–Cr green in green tea. Partial least squares discrimination (PLS-DA) was used for the qualitative analysis of Pb–Cr green, and the classification was 100% correct. The interval partial least squares (iPLS) regression combined with the successive projection algorithm (SPA) was proposed to select the characteristic wavenumbers for quantitative analysis of Pb–Cr green, and the least squares support vector machine algorithm (LS-SVM) was used to obtain the optimal model with *R_P_*^2^ of 0.864 and a root mean square error of prediction (*RMSEP*) of 0.291. The results showed that infrared spectrometry was feasible for the detection of Pb–Cr green in green tea. Kurrey et al. [36] used diffuse reflectance Fourier transform infrared spectroscopy (DRS-FTIR) for the rapid quantitative determination of antibiotics, ciprofloxacin (CIP), and norfloxacin (NOR) in poultry egg samples. The linear range of DRS-FTIR for the detection of CIP and NOR in poultry egg yolk was 0.05–0.50 ng mL^−1^, the limits of detection (LODs) were 0.032 ng mL^−1^, and the recoveries were 83.1%-102.3%. The method is simple, sensitive and suitable for high-throughput analysis of food samples. Gu et al. [37] studied the chemical morphological changes of squid immersed in different concentrations of formaldehyde using three infrared spectra (FTIR, SD-IR, and 2DCOS-IR). The predicted values of the constructed model were close to the actual formaldehyde concentration values in squid, with a prediction correlation coefficient of 0.9774, a root mean square error of prediction (*RMSEP*) of 6.08 and a limit of quantification of 15 mg kg^−1^. The method took 5 min to determine formaldehyde, while the HPLC method took 1.5 h to determine formaldehyde.

The analysis of the above studies shows that infrared spectroscopy is a good technique for the nondestructive detection of pesticide residues in fruits and vegetables, illegal additives in flour and green tea, and antibiotics in eggs. However, differences in the manufacturers of fruits, vegetables, and eggs from different origins and illegal additives used in the experiments may lead to the differentiation of the final models. Therefore, the establishment of a set of standardized and perfect data models for detecting illegal food additives, pesticides, and antibiotics could well improve the universality of the models and broaden the application of this technology.

### 4.2. Detection of Microbial Toxins

Toxins produced by microorganisms can be classified into bacterial toxins, actinomycete toxins, and toxins produced by fungi. Microbial toxins are an important factor in endangering human health. Therefore, it is very necessary to realize rapid detection of microbial toxins for people’s life and health and to ensure food safety.

Aflatoxin is a strong carcinogen, of which heterotoxin A is a precursor of aflatoxin B1 (AFB1) and can be used as an indicator of the presence of AFB1. Zheng et al. [38] developed a method for the detection of heterotoxin A in maize by near-infrared spectroscopy, using an extreme value gradient enhancement algorithm (XGBoost) combined with a support vector machine (SVM). A quantitative ranking and secondary quantitative ranking and secondary ranking methods were developed using the extreme value gradient enhancement algorithm (XGBoost) combined with SVM. The coefficient of determination *R*^2^ of the quantitative model was 0.9705, the root mean square error of prediction (*RMSEP*) was 3.567 μg kg^−1^, the relative prediction error (RPD) was 5.98, the accuracy of the ranking method was 90.32%, and a food safety classification system based on 0–110 μg kg^−1^ of trichothecene A in stored maize was established. Tao et al. [39] used visible-NIR spectroscopy to detect aflatoxin B1 in peanut kernels in the spectral range of 400–2500 nm. The overall accuracy was 88.57% and 92.86% with the threshold values, respectively. This method can provide an application basis for the subsequent detection of aflatoxin-contaminated agricultural products.

Shen et al. [40] investigated the quantitative and qualitative analysis of aflatoxins in brown rice by NIR and Mid-IR spectroscopic techniques combined with chemometrics, while aflatoxins B1, B2, G1, G2, and total aflatoxins were determined by the SMLR algorithm to select the characteristic wavelengths of sample spectra and establish PLSR models for NIR and Mid-IR spectral data, respectively. The NIR spectral model (*R* of 0.936–0.973, RPD of 2.5–4.0) and mid-infrared spectral model (*R* of 0.922–0.970, RPD of 2.5–4.0) both had good prediction accuracy. The main contaminant in peanut mold is aflatoxin, a toxin that poses a serious risk to life and health. Fumonisins (FBs) and zearalenone (ZEN) are naturally occurring mycotoxins in cereals and grains, both of which endanger human health. Tyska et al. [41] studied the quantitative determination of contamination levels of FBs and ZEN in Brazilian maize by NIR techniques, and the correlation coefficient (*R*), coefficient of determination (*R*^2^), and relative prediction error (RPD) of FBs were 0.809, 0.899 and 3.33, respectively. The correlation coefficient (*R*), coefficient of determination (*R*^2^), and relative prediction error (RPD) for ZEN were 0.991, 0.984, and 2.71, respectively. This method has proven to be a reliable alternative for the analysis of such toxins. Lin et al. [42] proposed a combined colorimetric sensor (CS) and visible–near-infrared spectroscopy method for the detection of volatile marker compounds in wheat, using the chemically reactive dye 8-(4-nitrophenyl)-4, et al., as a colorimetric sensor capture probe for volatile organic compounds and scanning CS-VNIRs spectral data to develop a Si-PLS model with a predicted correlation coefficient of 0.9387. Lim et al. [43] used NIR spectroscopy for the rapid nondestructive identification of Fusarium-infested barley in the 1175–2170 nm band with 100% accuracy using PLS-DA analysis for discriminative prediction. Dilek et al. [44] used the FTIR method to detect fumonisins due to *Fusarium niger* contaminated raisins and demonstrated that *Fusarium niger* contaminated raisins showed an increased absorbance in the characteristic bands at 1733 cm^−1^, 1736 cm^−1^, and 1708 cm^−1^, demonstrating that the FTIR spectroscopy technique is capable of determining fumonisins and other mycotoxins in agricultural products. Annalisa et al. [45] used Fourier transform near-infrared (FT-NIR) and Fourier transform mid-infrared (FT-MIR) spectra combined with chemometrics for the first time to detect deoxynivalenol (DON) in wheat bran samples. Standard normal variables (SNV) transformation, mean normalization, and other preprocessing methods were used to compare and fuse the processed spectral data. Partial least squares discriminant analysis (PLS-DA) and principal component linear discriminant analysis (PC-LDA) were used as classification methods with a threshold of 400 μg kg^−1^ DON. The overall recognition rate was 87–91% for FT-NIR and 86–87% for FT-MIR spectral ranges. Murat et al. [46] developed an artificial neural network (ANN) FTIR spectral analysis method for the rapid identification of *Bacillus cereus* and *Bacillus thuringiensis*. The spectral data were preprocessed, and the neural network model was constructed using the deep learning toolbox of MATLAB R2018b for feature selection. The model has a 100% recognition rate for the training set and a 99.5% overall recognition rate for the test set. This method can be used for the identification of *Bacillus cereus* in food as well as in soil samples.

The NIR spectra of microbial cell walls, cell membranes, and internal biomolecules are highly specific. Therefore, the use of NIR spectroscopy allows for the rapid identification and classification of different microorganisms. The method is simple to operate and is fast and nondestructive. Infrared spectroscopy has good application prospects for the detection of microbial contamination in food due to mold, bacteria, etc.

### 4.3. Detection of Other Toxic and Harmful Substances

The intake of trans fatty acid (TFA) is directly related to human health. TFA can cause a variety of diseases [47,48]. It is necessary to eliminate TFA from food products, of which hydrogenated oils are the main source of TFA. Khan et al. [49] used Fourier transform infrared spectroscopy combined with the second-order derivative method for a rapid determination of TFA content in selected Indian fast foods and hydrogenated fats. The TFA content in fast food products ranged from 1.57% to 3.83% of total fat, while in hydrogenated fats it ranged from 3.31% to 4.73%. The regression coefficient value of this method was 0.99503 with a standard deviation of 0.10247, and the test results were in good agreement with those from the gas chromatography flame ionization detector (GC-FID) method. Jiao et al. [50] used open-path FTIR for the first time to remotely detect volatile compounds in food products, and active and passive methods were used to obtain the volatile compounds released from white wine, vinegar, and grapes in the range of 5m. An atmospheric compensation of ethanol was combined with the PCA method by feature extraction to identify different brands of goods, as well as to assess the degree of food spoilage. Hui et al. [51] used a portable NIR spectroscopy system to determine the acidity of edible oils during storage and compared four variable selection methods (CARS, VISSA, IVSO, and BOSS) to optimize the NIR spectral data, and obtained support from vector machine models for different selection methods. The BOSS method yielded the lowest number of characteristic wavelengths. The number of variables obtained by this method is the lowest, and the SVM model built that was based on the optimized features of this method has the best prediction performance. The root mean square error of prediction (*RMSEP*) was 0.11 mg g^−1^, the coefficient of determination (*R_p_*^2^) was 0.92, and the relative prediction error (RPD) was 2.82. 

The formation of acrylamides has had a great impact on the potato processing industry. Smeesters et al. [52] used broadband reflectance spectroscopy (400–1700 nm) in combination with machine learning for the nondestructive detection of acrylamide in potatoes unsuitable for high-temperature processing. Linear discriminant analysis and extreme learning machine methods were used to classify acrylamide at different concentrations with an identification rate of 92%. Bisphenol S (BPS) is an alternative to Bisphenol A (BPA), and both are banned in food, both being toxic and harmful substances. Ullah et al. [53] determined the molecular vibrations of BPA and BPS by Fourier infrared spectroscopy with principal component analysis to investigate the correlation that exists between them. Ten frequencies are identified, suggesting the involvement of benzene rings in their toxicity or carcinogenicity. Docosahexaenoic acid (DHA) is an important ingredient in infant formula, and these long-chain polyunsaturated fatty acids (LC-PUFAs) are prone to oxidation. Daoud et al. used attenuated total reflection Fourier infrared spectroscopy and near-infrared spectroscopy to detect lipid oxidation in infant milk powder. A good correlation coefficient (R^2^ > 0.9) was obtained between the volatile content and the IR spectra. Near-infrared spectroscopy (NIRS) gave better results than ATR-FTIR. The prediction error of ATR-FTIR (18%) is higher than that of NIRS (9%). Therefore, near-infrared spectroscopy makes it possible to develop online detection for the production of infant milk powder.

In summary, it is feasible to use infrared spectroscopy for the detection of pesticides, veterinary drugs, antibiotic residues, illegal additives, mycotoxins, and other toxic and hazardous substances in food. However, due to the complicated variety of such toxic and hazardous substances, infrared spectroscopy still requires a lot of research on food safety detection. Table 1 summarizes the relevant literature on the above-mentioned infrared spectroscopy techniques for the detection of toxic and hazardous substances in food.

## 5. Summary and Outlook

In recent years, infrared spectroscopy has been intensively researched in the field of detection of toxic and hazardous substances in food. This paper reviews the latest progress in applying this technology for the detection of toxic and hazardous substances in food. Infrared spectroscopy has the advantages of rapid, high accuracy, and non-destructive in-line identification. It is expected to be an alternative or complementary method to existing food safety detection methods when combined with suitable chemometric methods. In addition, the combination of hyperspectral, infrared spectral imaging, surface-enhanced infrared spectroscopy, and other techniques can broaden the detection range and enhance the detection capability. Infrared spectroscopy, a nondestructive testing technique, has become the preferred testing method for more and more food products. At this stage, infrared spectroscopy has made great achievements in food safety detection, but there are still some difficult problems that need to be focused on with research at a later stage. The types of toxic and harmful substances in food are complex and difficult to detect. Novel substances will also be encountered in the future for infrared spectroscopy detection, which puts forward higher requirements on the performance of the instrument. High sensitivity, high accuracy, and good reproducibility are all key to achieving successful IR detection. Therefore, future research efforts should focus more on the following points:

(1) The infrared spectral fingerprint library of toxic and hazardous substances in the food industry has not yet been established. Thus, a complete, comprehensive, and standardized database of infrared spectral analysis models of toxic and hazardous substances in food is to be established for sharing resources and satisfying the needs for real-time online detection and analysis of such substances. Through standardized means, we can promote the development of infrared spectroscopy detection methods for toxic and hazardous substances in food and thus lay a good foundation for the application of infrared spectroscopy technology in food and agriculture.
(2) At present, most of the research on the application of infrared spectroscopy technology in the detection of toxic and hazardous substances in food at home and abroad is basic research done under laboratory conditions, which makes it difficult to meet the needs of the domestic food safety field. Therefore, the development of small and portable infrared spectrometers to meet detection requirements is another important development direction for infrared spectroscopy technology in the field of food safety. At the same time, this would assist in solving the technical bottleneck in instrument manufacturing, as well as for innovative training of instrument manufacturing personnel, and should be increased. Only in this way can we produce domestic infrared spectrometers that are better suited for the detection of toxic and hazardous substances in food.
(3) The development of chemometric methods. When conducting infrared spectroscopy on samples to be tested, both in terms of spectral preprocessing and establishing quantitative and qualitative analysis models, chemometric methods are needed. Understanding the current algorithms used in the detection of toxic and hazardous substances in food by infrared spectroscopy can help develop new algorithms and broaden the application of this technology in the field of food safety.
(4) The combination of infrared spectroscopy with advanced technologies, such as hyperspectral imaging, can yield richer feature information, including image information of external features and spectral information of internal features of food and agricultural products. This helps to more efficiently identify and quantitatively detect toxic and harmful substances in them qualitatively. As many of the food and agricultural products contain more moisture, the absorption peaks of water are stronger and may cover the absorption peaks of other substances. Therefore, infrared spectra are susceptible to the influence of moisture absorption peaks. The method of attenuated total reflection surface-enhanced infrared spectroscopy (ATR-SEIRAS) can effectively reduce the interference of solvent water on the signal to be measured [55,56], which can achieve highly sensitive and rapid quantitative and qualitative analyses.

With the continuous development of related theories and technologies, infrared spectroscopy will have more extensive applications in food safety and even biomedical fields.

## Figures and Tables

**Table 1:** Application of infrared spectroscopy for the detection of toxic and hazardous substances in food.

Detection Object	Spectral Method	Band Range	Analytic Procedure	Reference
Pesticide residues in strawberries	NIR	11,000–4000 cm^−1^	PLSR	[31]
Pesticide residues in the cabbage	Vis-NIR	350–2500 nm	LS-SVM	[32]
Talc powder in wheat flour	NIR	400–2500 nm	MIW-CCM-MSC-RBF	[33]
Azodiformyl in wheat flour	Vis-NIR	400–2500 nm	1st-SNV-RBF	[34]
Lead-chrome green in green tea	FTIR	4000–400 cm^−1^	PLS-DA LS-SVM	[35]
Fluoroquinolone antibiotics in poultry eggs	DRS-FTIR	4000–400 cm^−1^	Calibration Curve	[36]
Formaldehyde in squid	FTIR SD-IR 2DCOS-IR	2970–2920 cm^−1^ 1570–1520 cm^−1^ 1330–1280 cm^−1^ 1110–1060 cm^−1^	PLS	[37]
Aspamycin A in maize	NIR	400–2500 nm	SVM	[38]
Aflatoxin B1 in peanut kernel	Vis-NIR	400–2500 nm	PLS-DA	[39]
Aflatoxin in brown rice	NIR MIR	12,000–4000 cm^−1^ 4000–600 cm^−1^	LDA PLSR	[40]
Voltamycin and eryamenone in corn	NIR	1100–2500 nm	PLS	[41]
Mildew in wheat	Vis-NIR	350–1000 nm	Si-PLS	[42]
Fusarium species in barley	NIR	1175–2170 nm	PLS-DA	[43]
Voltamycin in raisins	FTIR	1800–800 cm^−1^	Normalization	[44]
Doxysaanenol in wheat bran	FT-NIR FT-MIR	10,000–4000 cm^−1^ 4000–350 cm^−1^	PLS-DA PC-LDA	[45]
*Bacillus cereus* and *Bacillus thuringiensis*	FTIR	3100–2800 cm^−1^ 1800–700 cm^−1^	ANN	[46]
Trans fat in Indian fast food and hydrogenated fats	ATR-FTIR	4000–800 cm^−1^	2st-LINEST	[49]
Volatile compounds in liquor, vinegar, and grapes	Open-path FTIR	4000–650 cm^−1^	PCA	[50]
Acid during storage of cooking oil	NIR	900–1700 nm	SVM	[51]
Acrylamide in raw potatoes	Vis-NIR	400–1700 nm	LDA ELM	[52]
Bisphenol S (BPS) and Bisphenol A (BPA)	FTIR	6800–400 cm^−1^	PCA	[53]
Oxidized DHA in infant milk powder	ATR-FTIR NIR	4000–400 cm^−1^ 1000–2500 nm	PLSR	[54]

## Data Availability

Not applicable.

## Abbreviations

ATR	Attenuated total reflection
ANN	Artificial neural networks
AFB1	Aflatoxin B1
BOSS	Bag-of-SFA symbols
CARS	Competitive adaptive reweighted sampling
CARS-PLS	Competitive adaptive reweighted sampling-partial least squares
DON	Deoxynivalenol
ELISA	Enzyme-linked immunosorbent assay
ELM	Extreme learning machine
FTIR	Fourier transform infrared
FBs	Fumonisins
GC-FID	Gas chromatography flame ionization detector
IPLS	Interval partial least squares
IVSO	Iteratively variable subset optimization
LDA	Linear discriminant analysis
LS-SVM	Least squares support vector machine
LC-PUFAs	Long-chain polyunsaturated fatty acids
MLR	Multiple linear regression
MLP	Multilayer perceptron network
MCR-ALS	Multivariate curve resolution-alternating least squares
NIR	Near infrared
PCA	Principal component analysis
PLS-DA	Partial least squares discriminant analysis
PLS	Partial least squares
PCR	Principal component regression
PLSR	Partial least squares regression
RBF	Radial basis function
RPD	Relative prediction error
*RMSEP*	Root mean square error of prediction
SVM	Support vector machine
SNV	Standard normal variate
SD	Second derivative
SIMCA	Soft independent modeling of class analogy
SVM-DA	Support vector machine-discriminant analysis
VISSA	Variable iterative space shrinkage approach
ZEN	Zearalenone
